# Language and culture in speech-language and hearing professions in South Africa: Re-imagining practice

**DOI:** 10.4102/sajcd.v68i1.793

**Published:** 2021-06-03

**Authors:** Katijah Khoza-Shangase, Munyane Mophosho

**Affiliations:** 1 Department of Audiology, Faculty of Humanities, University of the Witwatersrand, Johannesburg, South Africa; 2 Department of Speech Pathology, Faculty of Humanities, University of the Witwatersrand, Johannesburg, South Africa

**Keywords:** clinical curriculum; culture, diversity, epistemic disobedience, resource constraints, South Africa, speech-language, transformation

## Abstract

South African speech-language and hearing (SLH) professions are facing significant challenges in the provision of clinical services to patients from a context that is culturally and linguistically diverse (CLD) due to historic exclusions in higher education training programmes. Over 20 years postapartheid, little has changed in training, research, as well as clinical service provision in these professions. In line with the Health Professions Council of South Africa’s (HPCSA) SLH Professional Board’s quest to transform SLH curriculum and in adherence to its recently published Guidelines for Practice in a CLD South Africa, in this review article, the authors deliberate on re-imagining practice within the African context. They do this within a known demand versus capacity challenge, as well as an existing clinician versus patients CLD incongruence, where even the clinical educators, a majority of whom are not African, are facing the challenge of an ever more diverse student cohort. The authors systematically deliberate on this in undergraduate clinical curriculum, challenging the professions to interrogate their clinical orientation with respect to African contextual relevance and contextual responsiveness (and responsibility); identifying gaps within clinical training and training platforms; highlighting the influencing factors with regard to the provision of linguistically and culturally appropriate SLH clinical training services and, lastly, making recommendations about what needs to happen. The Afrocentric Batho Pele principles, framed around the concept of *ubuntu*, which guide clinical intervention within the South African Healthcare sector, frame the deliberations in this article.

## Introduction

Seabi, Seedat, Khoza-Shangase and Sullivan (2014) explored how speech-language and hearing (SLH), psychology and social work students experience living, learning and teaching in higher education in South Africa, revealing that there was a general feeling of dissatisfaction with the lack of transformation of the curriculum in these fields. In this study, factors negatively impacting the learning process were found to include high workload, English as a medium of instruction and limited access to ‘other’ resources (epistemological freedom). This study asserts that this limited access to epistemological freedom remains the prevailing condition within the SLH programmes in South African universities because of the consequences of the historic exclusion of black and African language-speaking candidates from South African higher education training programmes. This is because the epistemological frames that enjoy purchase in South African universities are ‘… largely still based on a Eurocentric, Western epistemology with minimal, if any, inclusion of Afrocentric epistemology and ontology’ (Khoza-Shangase & Mophosho, 2018, p. 3). The current authors published an article entitled ‘Language and culture in SLH professions in South Africa: The dangers of a single story (2018)’, framed around Novelist Chimamanda Ngozi Adichie’s (2009) TED talk ‘The danger of a single story’. This review article is a continuation of the process of confronting the dangers of ‘a single story’ started in 2018 by the current authors, which is a deliberate process for epistemic freedom and disobedience in the curriculum of these professions.

In a study of South African medical students by Matthews and Van Wyk (2018), findings indicated a strong desire for cultural competence in the curriculum to improve delivery of services and provide support to culturally and linguistically diverse (CLD) groups. Factors in epistemological access and language of instruction have been presented by the current authors in detail elsewhere (Khoza-Shangase & Mophosho, 2018), cautioning against a ‘single story’ in SLH professions. The Health Professions Council of South Africa’s (HPCSA) Board for SLH professions recently produced *Guidelines for Practice in a Culturally and Linguistically Diverse South Africa*, where one of its key recommendations is that training institutions ought to (HPCSA, 2019):

[*U*]se findings from locally relevant research to inform curricula; acknowledge and interrogate colonial influence in all its forms (power, being and knowledge) and move towards a repositioning of the professions to better serve all South Africans. (p. 15)

These guidelines are a major milestone in the tangible steps that the council has taken to ensure that clinical service provision within the South African context is Afrocentric and CLD competent. However, measures to ensure that the guidelines are adhered to by the South African SLH professionals are still not in place, but active enforcement of the guidelines by employers, professional associations through Continued Professional Development (CPD) initiatives and the council in its annual CPD compliance audits of practitioners can facilitate compliance.

### Speech-language and hearing practice in the culturally and linguistically diverse South African context

Khoza-Shangase and Mophosho (2018) suggested recommendations for the South African context that as part of transformation and decolonisation: (1) the SLH professions need to actively engage with the national calls to Africanise institutions and clinical service delivery, (2) clinical care changes that are contextually relevant and responsive, (3) research focus on local needs and issues for local benefit, (4) clinical focus that will allow for ‘next practice’ and not just ‘best practice’, (5) language policy that respects that many people speak several languages and so teaching and learning (and clinical service provision) in only English and/or Afrikaans creates challenges, which need addressing and so on (p. 6). These arguments are anchored in the Constitution of the Republic of South Africa (1996) and are supported by evidence from Statistics South Africa (2018), which estimates the country’s population to be at 57.7 million. This population consists of a diversity of cultures, languages, religions, nationalities and ethnicities, with 80.2% of people being black Africans – a majority of which speak isiZulu (23.8%) as a home language, with English being spoken as a home language by only 9.6% of the population.

This evidence highlights the need for re-visiting and re-imagining practice in SLH to come in line with lived context to ensure positive patient outcomes. This would also be in line with the Universal Declaration of Human Rights (United Nations, 1948), the 2003 National Language Policy Framework and the 2012 *Use of Official Languages Act* as outlined by the South African Department of Arts and Culture (2003). The Department of Health (DoH) standards for accreditation in undergraduate programmes in SLH state that curriculum for academic and clinical education ought to ‘ensure that provision of services to clients or patients is not compromised where the clinician does not speak the client’s or patient’s language’ (DoH, 2014, p. 7). Furthermore, on the African continent, it behoves local professions to move away from Euro-centric thinking and embrace decolonial practices.

Decolonisation entails ‘deconstruction and reconstruction’, that is (Ndlovu-Gatsheni, 2018):

[*D*]estroying what has wrongly been written – for instance, interrogating distortions of people’s life experiences, negative labelling, deficit theorising, genetically deficient or culturally deficient models that pathologizes [*sic*] the colonized [*sic*] other – and retelling the stories of the past and envisioning the future. (p. 38)

The intellectual landscape of SLH programmes in training institutions could adopt the *ubuntu* worldview. This worldview, ‘… recognises the importance of others, of history, of context and community in the formation of one’s identity and the interdependent relations between individuals and collectives’ (Oelofsen, 2015, p. 141).

Sufficient evidence exists to support the positive outcomes linked to health interventions conceived and implemented, taking careful cognisance of patients’ language and culture. Matthews and Van Wyk (2018) asserted that integrating culture and language in teaching and learning may be a facilitating factor in developing cultural competence for medical students and improving healthcare and outcomes in the South African context. This is supported by evidence on global health that indigenous groups worldwide tend to have worse health outcomes than corresponding non-indigenous populations (Anderson et al., 2016). Flood and Rohloff (2018) stated that global health interventions planned and delivered using indigenous languages are likely to be more successful, that groups that are not part of the dominant culture have less positive health outcomes than the dominant communities, with language presenting as a barrier to healthcare delivery, and that real indigenous language sensitivity includes not only the use of interpreters and translators, but also the creation of language-oriented programmes from the initial stages. Sue and Sue (2003) maintained that patients seen by healthcare providers whose racial and linguistic background matches their own continue to attend their treatment sessions and maintain treatment plans for longer duration; and this speaks directly to adherence, which is an important factor in SLH intervention success.

Kathard and Pillay (2013, p. 85) recommended that speech-language therapists acquire political consciousness, which implies that they ‘… be tolerant and accepting of being disrupted in order to challenge and change historical, taken-for granted practices’. In this regard, Pillay and Kathard (2018) recommended using the Equitable Population-based Innovations for Communication (EPIC) framework, which aims to decolonise the SLH profession by developing services that are equitable and which meet the needs of underserved populations. Sue and Sue (2003) advocated for racial-cultural competence, which obligates therapists to be able not only to interpret the knowledge that they have about their client’s culture, but also the ramifications of race. Subsequently, acquiring raised awareness of racial aspects of diversity is important because diversity is not only about culture, gender, socio-economic status, disability or language but also includes race. Frequently, white individuals regard racial-cultural differences (e.g. expressiveness of verbal and non-verbal communication of African people) as a form of deviance (Sue & Sue, 2003), and this misinterpretation has serious ramifications for professions dealing with communication disorders.

Clinicians ought to be cautious about these misconceptions, as they may lead to misdiagnosis. Oelofsen (2015) advised white academics in South Africa, such as herself, to move away from their identity of advantage and embrace a hybrid identity, which has values and concepts from the traditional African concepts. She advocates that white academics ought to learn an African language as part of performance appraisals. Universities ought not to stay silent when injustice unfolds; they are instead obliged to speak up, talk back and push the boundaries to become relevant to the context (Bell, Canham, Dutta, & Fernández, 2019). If university personnel adopt such strategies, they facilitate the development of multicultural competence within their spaces, which leads to lifelong practice outside. It is of importance to note that for multicultural competence to be lifelong practice, it is essential that it should be developed initially as part of clinical training (Falender, Shafranske, & Falicov, 2014b).

The SLH professions both in South Africa and more globally must accept that sensitivity to their patients’ cultural and linguistic context – in the form of critical diversity literacy – is pivotal. The ultimate point of achieving critical diversity literacy is being conscientised at a cognitive and affective level. The SLH professions in South Africa need to be conscientised to the fact that the burden of disease and structural inequalities affect the poor, which, because of apartheid oppression, are mostly the black people. These constitute the majority patients in the public hospitals (Kathard & Pillay, 2013). As asserted by Freire (1998), conscientisation requires both reflection *and* action, with action being fundamental in dismantling the status quo as key to the SLH professions in South Africa. Stein (2010) reiterated that symbolic and material values of hegemonic identities do become established in social relations. Healthcare professionals need to be able to identify the ways in which issues such as whiteness, masculinity, heterosexuality, able-bodiedness and middle-classness are accepted as the norm and are reproduced in contexts such as healthcare. Furthermore, it becomes highly challenging to change mindsets in society and shift the status quo. Ethnic disparities in obtaining medical care are a good example in the South African context.

As part of its mandate to ‘protect the public’ and ‘guide the professions’, in its concerted and sustained effort to transform the SLH professions in South Africa, the HPCSA (2019) SLH Professional Board released *Guidelines for Practice in a Culturally and Linguistically Diverse South Africa.* These guidelines propose five main principles to be adhered to by the SLH professions. Principle 1: contextual relevance as an overarching philosophy for more relevant practice that will lead to more effective management of communication difficulties (to take cognisance of the processes and protocols adopted in clinical practice); Principle 2: focus on assessment and intervention (to practise epistemic disobedience in terms of what knowledge and which knower is the respected source); Principle 3: importance of local knowledge and calls for a shift in how the profession values it (Khunou, Canham, Khoza-Shangase, & Phaswana, 2019) (to examine the ‘how’ of clinical training within the South African context); Principle 4: focus on clinical training (remaining vigilant in continued professional development of critical consciousness); Principle 5: lifelong development of critical consciousness. The current authors believe that these principles, if adhered to and practised, would not only lead to positive SLH services outcomes but would also contribute significantly towards the transformation goals of the SLH programmes to make them more Afrocentric. This would, therefore, require enforcement and accountability – as the voluntary, optional ‘goodness of the heart’ approach does not seem to have yielded tangible outcomes over two decades after the democratic dispensation. This article is a clarion call to the HPCSA SLH Board, Departments of Health and Education (as largest employers) and SLH training institutions to ensure that translation of these principles into practice occurs.

On the importance of epistemological and language diversity access, for example, Southwood and Van Dulm (2015) argued that even though more speech language therapists who can work in African languages are entering the profession now than before, the lack of access will continue to make it difficult to disentangle children who are speakers of African languages and who are typically developing but still in the process of acquiring English (or Afrikaans) from those who have underlying language impairment. They lament that this is because such a differential diagnosis requires knowledge of the status of the child’s language skills in his or her first language, which most clinicians are not privy to. They therefore conclude, similar to current authors, that recruiting first or other language speakers of African languages into SLH training programmes promises to change levels of service delivery more than specialised training or maturation as an SLH practitioner. These authors, as argued by Khoza-Shangase and Mophosho (2018), suggested that such recruitment and the appropriate training of African language speakers into SLH professions ought to be performed concurrently with development of linguistically and culturally appropriate assessment and remediation materials if efficacious contextually relevant research evidence-based assessment and intervention is to be provided (Southwood & Van Dulm, 2015). Pascoe and Smouse (2012) also argued that:

[*C*]linicians within the SLT profession have an ethical responsibility to effectively assess and manage their clients in the client’s first language, even where a language mismatch between client and clinician exists. (p. 471)

### Gaps in the South African speech-language and hearing clinic

The South African context shows significant mismatch between SLH practitioners and the population they serve with regard to language (English and/or Afrikaans) and culture (mostly Western). This incongruence creates a barrier to provision of efficacious clinical services and prevents growth and development of the professions. With only approximately 5% of HPCSA registered practitioners being black African language speakers – in a country that has over 70% of its population speaking an African language as their first language – an obvious gap exists. This gap is not localised to the clinical service provision by graduates but exists in the staffing profile of academics and clinical supervisors and/or educators in universities and clinical training platforms. This reality means that even African language-speaking students who enrol into South African training programmes receive training that is not ‘Africanised’. Consequently, they do not necessarily graduate in a better professional position than non-African language speakers. This is well described by Letsoalo and Pero (2020), who pointed to the *white gaze* and its persistent influence over the curriculum.^1^

**FIGURE 1:**
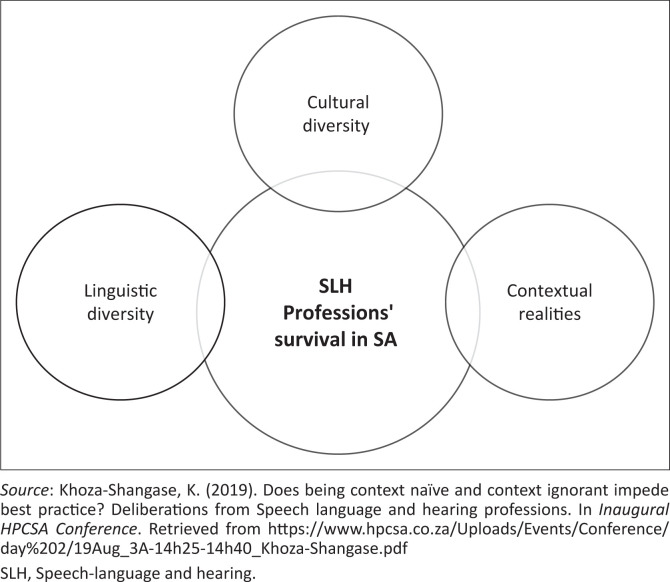
Tripartite threat to the survival of speech-language and hearing professions in South Africa.

If the demographic profile of teaching and research staff is not diversified in order to reflect the contextual profile, there is limited, if any, epistemological access, which in turn influences the beliefs held and approaches adopted about communication disorders and their assessment and management. This extends to the development of assessment and intervention resources that are linguistically and culturally appropriate over and above the translation and adaptations of resources, which has its own limitations and weaknesses for this context. As a result of the very nature of the SLH professions to assess and treat language and communication pathology in patients, this gap cannot continue to be ignored (Khoza-Shangase & Mophosho, 2018; Mophosho, 2018; Mdlalo et al., 2019; Pascoe et al., 2017). In fact, Khoza-Shangase (2019) presented a tripartite threat to the helping professions (SLH) professions in South Africa (Figure 1), as including ignorance and naivety about the influences of linguistic and cultural diversity over and above its contextual realities, such as burden of disease, resource constraints, poverty and inequality. Moreover, it is pivotal for clinical educators to acknowledge that their primary goal is to facilitate: ‘… thoughtful, systematic reflection about the contextual and multicultural factors affecting the clinical and supervisory relationships’ (Falender et al., 2014b, p. 60).

### Influencing factors to the provision of linguistically and culturally appropriate speech-language and hearing clinical training services

The current authors believe that a key challenge in achieving linguistically and culturally appropriate SLH clinical services in South Africa is the knowledge base, theory, research evidence (academic curriculum) that fails to reflect diverse knowledge, particularly to the exclusion of African epistemologies (Khoza-Shangase & Mophosho, 2018). Reeler (2015) argued for a curriculum that ‘deliberately aims to interrupt the usual hierarchy of knowledge’, calling for:

… content which consciously aims to engage with African epistemologies, be it through teaching postcolonial theory; deconstructing dominant canons or worldviews; using African examples, texts and contexts; correspondent examples or theories from other parts of the so-called majority world countries; or a pedagogy that uses African languages as learning resources. (p. 1)

**FIGURE 2:**
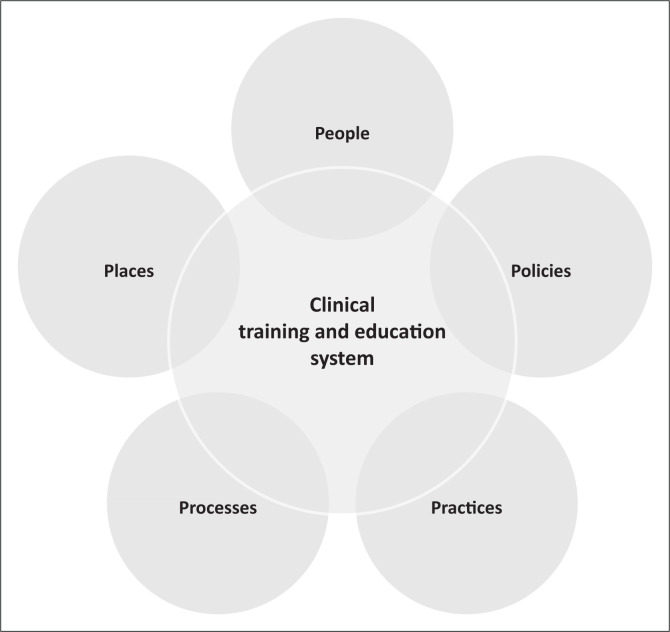
The 5 Ps of influencing factors towards decolonial speech-language and hearing professions in South Africa.

This study is grounded in postcolonial theory that addresses the current political, aesthetic, economic, historical and social impact of European colonial rule around the world (Elam, 2019).^2^ Although postcolonial theory takes many different shapes and interventions, the authors support Elam’s (2019) assertion that all the variations incorporate an important contention, namely that our understanding of the world is unfeasible outside of relating it to the history of imperialism and colonial rule, in terms of Europe’s colonial encounters and oppression around the world. This is argued here to be the case in SLH within the African context. These influencing factors within the clinical training and training platforms in the South African context, as reflected in Figure 2, are further impacted by several other factors, which can be grouped into five aspects: people, places, practices, processes and policies, guided by *National Center for Culturally Responsive Educational Systems* and invitational education theory (Purkey, 1999), which the current authors adapted for the current review article.

In terms of *people*, the current cohort of students, including clinical supervisors and/or educators, as well as the current combination of clinicians and patients, are mismatched both culturally and linguistically. The current cohort of clinical educators is not reflective of the demographic profile of the country, and consequently their linguistic and cultural competence and awareness of the context are limited. Furthermore, evidence of restricted training in African cultural and linguistic knowledge, for example, cultural humility, is seen to be generally lacking in the SLH curriculum. Limited tuition in courses such as African Anthropology and Sociology, over and above African languages, has been observed in all training programmes. These weaknesses exist in the context of a student body that remains largely non-linguistically and non-culturally diverse, with small numbers of African students assimilating into a distinctly non-African curriculum.

Firstly, as far as *practices* as an influencing factor are concerned, the highly structured curricula of the various programmes, in line with the HPCSA regulations, seem to be to the exclusion of CLD – unless there is a specific staff member interest. This leads to optional input in different programmes and unequal access and competency by students from different institutions. Secondly, the current authors argue that there is generally limited critical consciousness in clinical practice, with supervisors and/or educators not being aware of the influence of factors such as power, cultural capital, etc., in clinical provision and clinical training. We need to recognise that power and culture intersect within communicative interactions. According to Hyter and Salas-Provance (2019), power can be exerted using different measures, such as structural violence, physical violence, symbolic violence, manufactured consent and organisation of work. Speech-language and hearing practices ought to avoid all these power differentials, especially symbolic violence, where benefits of one group depend on the disadvantage or deprivation of another group.

There is a need for programmes to deliberate on culturally responsive pedagogy and practice with training provided as part of clinical educators’ training. Thirdly, there is unabated use of CLD inappropriate resources, where it is deemed acceptable to simply translate resources without following structured protocols or considering the adaptations and expertise of translators. Barratt, Khoza-Shangase and Msimang (2012) stated that translating and adapting a test are critical processes that need to consider the personal characteristics of individuals translating the test, which will influence how the test is translated. These authors raise caution about, for example, any inappropriate use of vocabulary or sentence structure, which they argue are some of the factors that may dilute the complexity of the translated material.

The current authors feel strongly that translations and adaptations ought to be performed by expert speakers of the target language, preferably from an academic linguistics department, who would understand the importance of maintaining the validity and usefulness of the test measure. Furthermore, current authors recommend that where translations and adaptations are performed, specific documented protocols ought to be followed, over and above simply translating and piloting the materials. Specifically, protocols followed ought to include translating and adapting the measure, reviewing the translated or adapted version of instrument, adapting the draft instrument based on the comments of the reviewers, pilot testing the instrument, field testing the instrument, standardising the scores and performing validation research (Geisinger, 1994). Peña (2007) aptly asserts that during translation:

[*T*]ypically, the translation process focuses on ensuring linguistic equivalence. However, establishment of linguistic equivalence through translation techniques is often not sufficient to guard against validity threats. In addition to linguistic equivalence, functional equivalence, cultural equivalence, and metric equivalence are factors that need to be considered… (p. 1255)

The current culture of using *planisa* as a lingua franca in CLD resources ought to be called into question, as it not only negatively impacts the accuracy and efficacy of clinical practice, but also infringes on patients’ rights. Mophosho (2016, p. 162) describes *planisa* as ‘… they get on and get by in the hospitals through their improvisation and the teams’ self-initiated action’.^3^ Furthermore, the use of untrained interpreters during assessments and intervention with limited use of cultural brokers forms part of the *planisa-*linked factors influencing the provision of linguistically and culturally appropriate SLH clinical training services within the South African context.

South Africa has a strong constitutional, legal and policy dispensation that protects patient rights. At the policy level, implementation and monitoring of the Language Policy in the DoH and Batho Pele (People First) principle; translating existing policies and regulations around CLD in healthcare into practice; confronting the use of *academic freedom* by training institutions to defend lack of social accountability and responsibility around CLD matters; raising and sharpening political will and government mandate around CLD; as well as confronting the silence around clinical practice without CLD competence in the ethical codes of conduct all present significant challenges. Councils such as the Council of Higher Education (CHE) and HPCSA, involved in quality assurance of higher education training, have a significant role to play here. Their failures in this regard illustrate the disjuncture between the universal aspiration of human rights as norms and the complexities arising in their implementation.

As far as *places* (physical environment in which people interact), as a factor, are concerned; it is a context where many of the speech language therapists and audiologists do not speak the language of their clients, consequently limiting their work. The paradox remains a structural mechanism in contemporary South African public health system that does not seem to be responsive to the needs of the citizens. Again, the context is also limited by existing interpreter resource challenges. Given that cultural and linguistic diversity has a profound effect on the ways in which families and professionals inter-relate cross-culturally and participate together in treatment programmes, the DoH ought to invest in finding trained interpreters to assist healthcare providers and patients in government institutions. In public settings where there are no mediated or interpreter services, the objectives of the National Language Policy Framework are certainly contravened.

Lastly, as far as *processes* (systematic series of actions directed to some end) are concerned, Penn, Mupawose and Stein (2009) attributed the lack of preparedness of speech language therapists and audiologists in a South African health context to professional, technical, systemic, managerial, interpersonal and ethical challenges. These can be attributed to a variety of complex factors, including gaps in resources including research, culturally appropriate intervention tools and relevant human resources.

## Solutions

**FIGURE 3:**
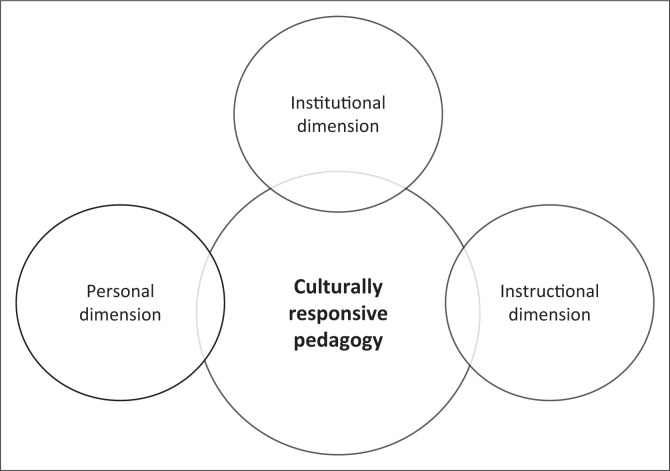
Clinical training through culturally responsive pedagogy (and practice).

Falender, Shafranske and Falicov (2014a) observed culturally responsive pedagogy in clinical training to include personal dimensions, institutional dimensions and instructional dimensions, as depicted in Figure 3.

In line with Figure 3, which defines culturally responsive pedagogy as requiring attention to the personal, institutional, as well as instructional dimensions; the aforementioned gaps and influencing factors, as numerous as they are, are not insurmountable. A multipronged approach is required, where a number of factors are simultaneously addressed as possible solutions to the current CLD conundrum facing the South African SLH professions. Key to this is the transformation of students and staff demographic profiles to become reflective of the country’s CLD profile. Speech-language and hearing professions need to also understand change processes in public policy implementation within the institutional dimension (Wylie, McAllister, Davidson, & Marshall, 2013).

Within the personal dimension, cultural competence humbles the practitioner and stimulates cultural humility. In addition, it enhances patient care, as the understanding of patient belief systems becomes integral to their healthcare (Juarez et al., 2006). This leads to cultural humility, which is defined as a lifelong learning process that incorporates openness, power-balancing and critical self-reflection when interacting with people for mutually beneficial partnerships and institutional change (Tervalon & Murray-Garcia, 1998).

At an instructional level, SLH departments need to have a student-centred vision and mission statement, commitment to diversity, provide culturally and linguistically appropriate services, with equitable and supportive learning environments, such as the conceptual framework for Responsive Global Engagement (Hyter & Salas-Provance, 2019). Furthermore, in relation to the clinical component, the current researchers recommend that clinicians collaborate with other disciplines, such as Sociology and Anthropology, and include clients and their families to understand the cultural history of persons on their caseloads. The worldview, cultural assumptions and practices of the client are considered in the clinical service by incorporating them into assessment and intervention plans (Hyter & Salas-Provance, 2019).

Research into CLD knowledge base and clinical practice, leading to development of CLD resources that are evidence based, is also paramount. Furthermore, taking a social action approach would be pivotal. This is the highest level on the Banks’ (1989) model, which involves students taking decisions and actions on important social issues. This can happen if training institutions have the political will and if programme leadership lobby not only for policy formulation around clinical care and clinical practice more generally but also specifically for training purposes in training platforms. For example, training institutions can play a major role in influencing the inclusion of official and trained interpreters in staff establishments such as hospitals, which serve as training platforms. Furthermore, clinical educators need to be trained to engage diverse and/or Afrocentric minds when consuming literature or clinical training sources, so as to ensure that their teaching and assessment approaches are contextually relevant. In addition to this engagement of diverse minds, clinical educators need to be assisted in developing multicultural competence, where their awareness of influence of culture on clinical presentation and clinical interaction is raised and where their awareness of the influence of culture on the supervision process is highlighted. All these speak about culturally responsive (and competent) pedagogy (Falender et al., 2014a) and cultural humility (as counter to ethnocentricity or white privilege) during the supervisory – supervisee/therapist – client dynamics.

Clinical supervision of SLH students that are CLD competent requires *commitment to social justice and advocacy*, where such a commitment underlies the attitude necessary for effective diversity and multicultural competence (Vasquez, 2012). Those involved should have a clear understanding of environmental contributions to human development, including pathology. Therefore, the curriculum needs careful interrogation in order to be CLD sensitive and competent. This is for both assessment and intervention with development of guidelines for clinical educators and clinician students forming part of the core clinical curriculum. The core curriculum should include diversity sensitivity programmes such as those provided by the People for Awareness on Disability Issues (PADI), an academic service-learning programme for first-year SLH students with disability awareness workshops run over the span of one semester specific to language and culture. These should not be optional as they currently are in most programmes. These types of service-learning projects also enhance civic responsibility in students because they focus ‘… on meeting both communities’ needs and learning objectives; it emphasises critical thinking to improve skills and civic responsibility’ (Pillay & Ramkissoon, 2020, p. 1075). Linguistic and cultural consciousness and critical diversity literacy should form part of the CLD enhancing tuition in SLH programmes.

## Way forward and conclusion

It is important that when South African SLH professionals deliberate on transformation of the profession, they should also face the decolonisation of the curriculum by introducing political consciousness and critical literacy diversity. Political consciousness in the curriculum and training is not just about learning to pass, but it teaches students how to respond to cultural context, foster connection with patients or clients and invest in relationships with the communities.

The SLH professions in South Africa need to show greater recognition of the urgent need to both examine and re-imagine SLH clinical training, which will have a positive effect on clinical outcomes and will inform academic curriculum. Training programmes need to identify environmental and instructional elements of culturally (and linguistically) responsive training and training platforms so as to ensure that these elements are optimised for their students’ clinical training. Training institutions need to deliberate on and define culturally (and linguistically) responsive pedagogy and hold themselves accountable to it with education and training regulating bodies, including this as part of institution evaluation and accreditation processes. This includes identifying features of culturally (and linguistically) responsive pedagogy – as recommended by the National Center for Culturally Responsive Educational Systems (NCCRESt, n.d.).

The South African undergraduate clinical curriculum needs to undergo reform, where standards followed are international, but Afrocentric with SLH training programmes from all South African universities understanding that they have a moral obligation to actively engage with and address the legacy of apartheid in all their teaching and learning activities (including their CPD offerings). Student clinicians and qualified clinicians ought to demonstrate an understanding of their client’s challenges, causes of the problems and work collaboratively with their clients in identifying ways to overcome them. Supervisory relationships should have a framework that has multicultural perspectives and diversity mindfulness; in addition, it should consist of an explicit antiracism and anti-oppression standpoint (Porter, 2019).

As far as the CPD offerings are concerned, professional associations/bodies have an important role to play as accreditors of CPD events and CPD providers themselves, in making sure that CLD activities are also prioritised. The South African government must be lobbied to address the CLD-related challenges in healthcare facilities, which serve as clinical training platforms, as state responsibility.

In re-imagining practice, the current authors have presented a number of suggestions, including decolonisation of the SLH professions with responsibility to context through best practice-driven clinical training that is contextually relevant and responsive. This is important if the SLH professions provide efficacious services within the National Health Insurance where universal health coverage is the goal. If the status quo remains, where there is a lack of demand-driven engagement, South African SLH practitioners will continue to rely on the *single story,* which impedes provision of effective intervention. This will continue to maintain the cycle of exclusion, with patients or clients entering the healthcare system but exiting without receiving effective treatment or care, because language and culture remain barriers in an environment where communication pathology is the presenting disability. Social structures of clients ought to be considered contextual factors when planning SLH assessments and interventions. For the SLH professions to be effective and relevant within the South African context, practitioners need to deal with the unequal relations of power and strive to maintain equality in these relations with those they serve – both in clinical training and clinical service provision. The authors have deliberately not included a template or a case example to illustrate implementation of the suggestions they have provided. This series remains a call for independent critical deliberations and engagements with the SLH practice, which must leave room for independent creativity in re-imagining SLH practice within the African context, thereby allowing us to carve out *next practice* for our context.

## Footnotes

1 *White gaze* is a popular term in the discourse of African-American literature, which captures black lived experience in white spaces, where whiteness imposes itself as a lens for knowledge, knowing and the knower (Khunou et al., 2019).

2 Other anti-colonial positions such as African Humanism, black Marxism, social theory also pertain.

3 Phakathi (2013) describes *planisa* as a type of *Fanakalo* (mining lingua franca), collaged together from various languages for miners to help them problem-solve day-to-day challenges.
